# Challenges in Diagnosis and Treatment of Mandibular Florid Osseous Dysplasia: A Case Report

**DOI:** 10.7759/cureus.74981

**Published:** 2024-12-02

**Authors:** Mohammed Essioti, Hind Boukaaba, Anouar Titou, Dounia Kamal

**Affiliations:** 1 Department of Reconstructive and Maxillofacial Surgery, Hassan II University Hospital of Fez, Fez, MAR; 2 Department of Reconstructive and Maxillofacial Surgery, Sidi Mohamed Ben Abdellah University, Faculty of Medicine, Pharmacy, and Dentistry, Fez, MAR

**Keywords:** cementum, conservative, dysplasia, florid, mandible

## Abstract

Florid osseous dysplasia (FOD) is a rare, benign fibro-osseous lesion primarily involving the mandible and maxilla, with a higher prevalence in middle-aged women of African or Asian descent. This article presents a case of mandibular FOD complicated by secondary infection, emphasizing clinical presentation, diagnostic challenges, and treatment strategies. The unique radiographic features of FOD necessitate careful differential diagnosis to prevent misdiagnosis with other jaw lesions. Conservative management, including infection control and surgical debridement, along with long-term follow-up, is recommended. This case underscores the importance of a cautious diagnostic approach and consistent follow-up for effective FOD management, to prevent further complications, especially in symptomatic cases.

## Introduction

Florid bone dysplasia (FOD), formerly known as florid cemento-osseous dysplasia, is a benign fibro-osseous lesion that usually occurs in the maxilla and mandible. The condition often affects middle-aged women, with a higher prevalence in people of African or Asian descent [1,2].

It is classified into three types according to radiographic features and lesion distribution [1], (1) Periapical (surrounds the periapical region of teeth and is bilateral), (2) Florid (sclerotic symmetrical masses), and (3) focal (single lesion) cemental dysplasia.

FOD is usually asymptomatic and is identified incidentally on routine radiographs. It presents as multiple radiopaque masses within a peripheral radiolucent rim located in two or more dental hemiarcades. However, infection or tooth extraction in the affected area may lead to symptoms (such as the appearance of pain, pus or exposure of a sclerotic, yellowish, avascular calcified mass in the oral cavity) and complicate management [3]. This report describes the case of a patient in whom florid cement-osseous dysplasia was diagnosed on the basis of clinical, radiographic and histological findings.

## Case presentation

**Figure 1:**
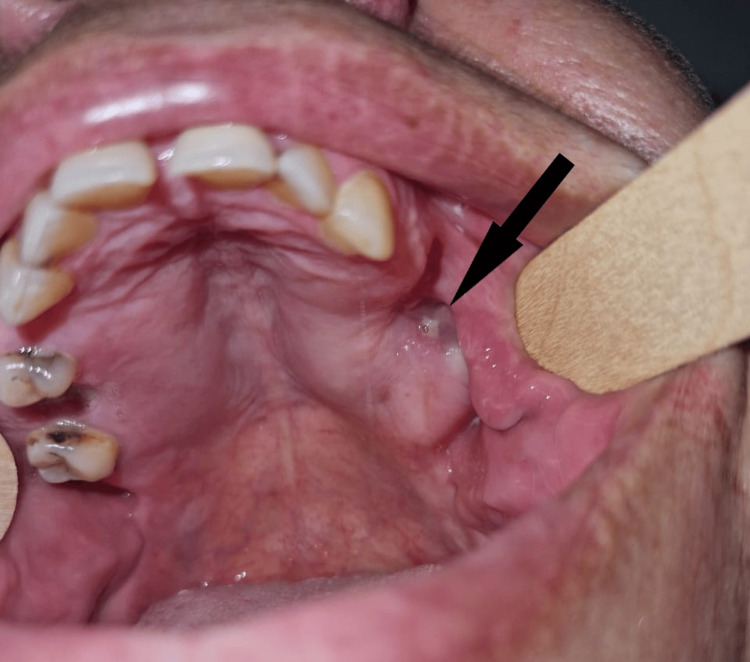
Intraoral examination image Intraoral image showing purulent discharge in the upper left molar region (black arrow).

The patient is a 49-year-old woman with no significant medical history. She presented with pulsatile pain, swelling and bleeding of the left mandibular mucosa that had been present for two years. Bilateral mandibular swelling was noted during the extraoral examination. The intra-oral examination also revealed calculus and gingival inflammation in the region of the lower incisors and canine. Teeth 14, 18, 24, 25, 26, 28, 36, 37 and 46 were missing, but the other teeth were in place. A mucosal ulceration with a hard, watery, exposed mass was found in the region of the left mandibular molars. It was accompanied by a purulent discharge on palpation (Figure 1).

**Figure 2:**
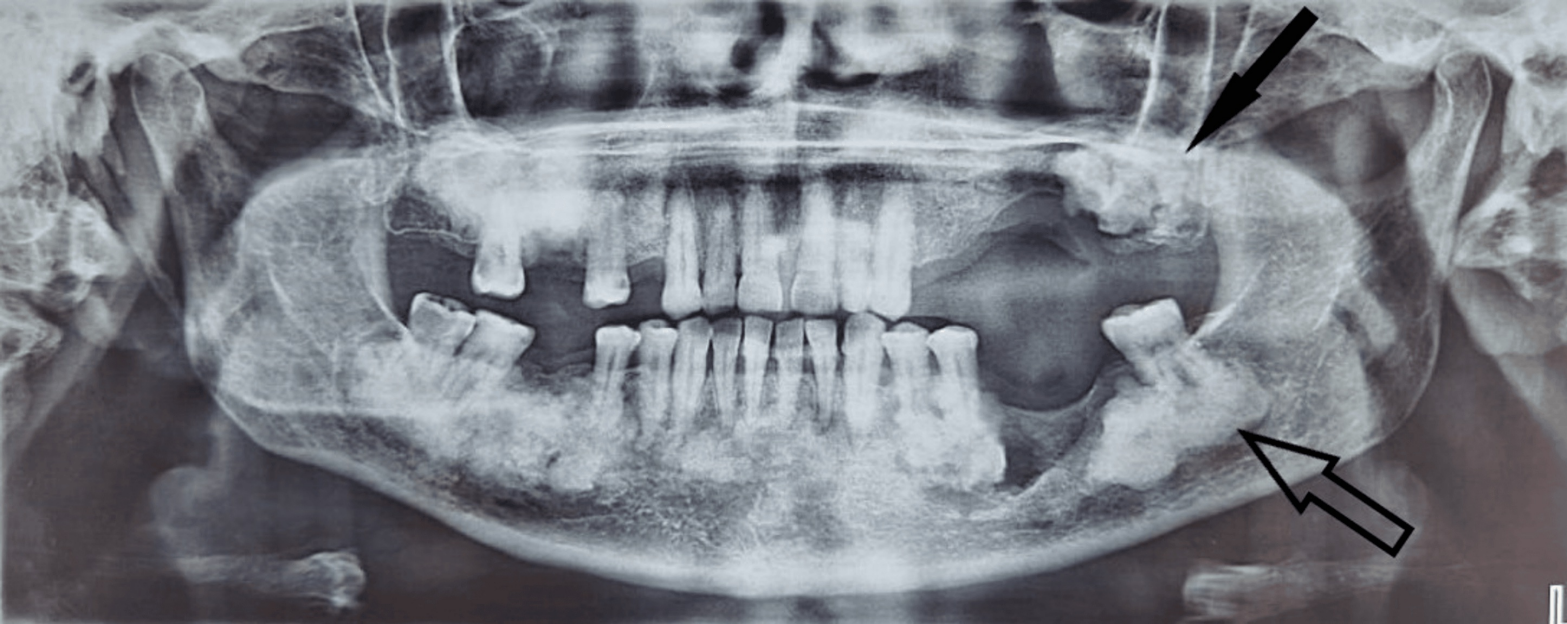
Pre-operative orthopantomogram image Pre-operative orthopantomogram reveals a radio-opaque lesion with a radiolucent margin localized on the periapical area of tooth #38 (hollow arrow) and in the edentulous area corresponding to tooth #28 (solid arrow).

Panoramic radiographs (Figure 2) showed amorphous radiolucencies in the posterior edentulous regions and in the periapical areas of the lower incisors and canines. The lesion extended beyond the alveolar crest and was surrounded by a radiolucent zone that extended into the left mandibular canal.

**Figure 3:**
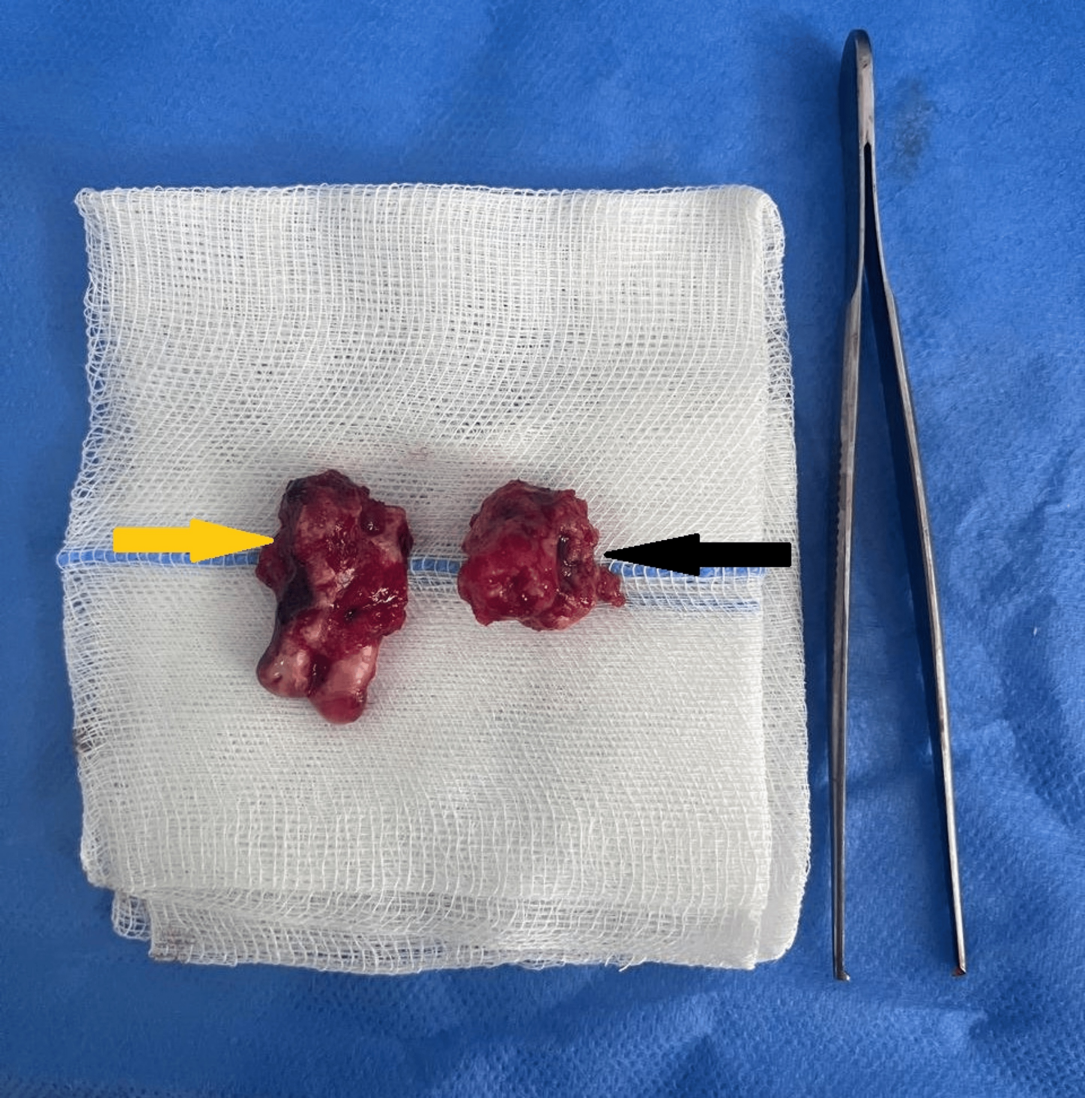
Extracted teeth image Extracted teeth (yellow arrow) with associated lesion (black arrow).

Possible diagnoses included osteitis, focal cemento-osseous dysplasia, FOD and peripheral ossifying fibroma. The initial treatment consisted of antibiotics to treat the infection, followed by surgical excision of the infected masses *(*measuring 1.25 x 2.08 cm*) *and extraction of the impacted teeth under general anaesthesia (Figure 3). This approach is consistent with the standard of care for infected FOD lesions and aims to remove the source of infection while minimising damage to surrounding structures.

The final diagnosis was confirmed by histopathological examination of the surgical specimen, which showed irregular cementum-like masses within a fibrous stroma, characteristic of FOD. The patient was followed up with regular clinical and radiographic assessments to monitor the status of the remaining lesions and to ensure that they remained asymptomatic. At the time of writing this report (one year after surgery), no recurrences have been observed.

## Discussion

Florid bone dysplasia (FOD) was first described by Melrose et al. [4] and renamed by Waldron CA [5] with the addition of the term ‘cemento-osseous’ because of the resemblance of the masses found to cementomas. It is often discovered incidentally due to its generally asymptomatic nature and is seen almost exclusively in women of African descent and of middle to advanced age. However, some cases in patients of Asian origin have been reported by Loh and Yeo [6].

A striking feature of the lesions is that they are located bilaterally, often very symmetrically, affecting the mandibular and/or maxillary molars and premolars, although unilateral involvement is not unusual. The majority of patients are edentulous or partially edentulous at the time of initial presentation [5].

Diagnosis of FOD is difficult due to radiographic overlap with other conditions, particularly when symptoms develop as a result of secondary infection. Radiographically, FOD presents as multiple dense, well-defined radiopaque lesions that progress to sclerotic masses [7]. Although histopathology provides a definitive diagnosis, a biopsy is generally avoided due to the risk of infection and pathological fractures resulting from the avascularisation of the dense cementitious tissue.

Pathologies such as Paget's disease, ossifying fibroids, and chronic osteomyelitis must be excluded. Paget's disease usually affects elderly patients and has systemic manifestations, whereas ossifying fibroids are typically solitary, expansile, and asymmetric. Chronic osteomyelitis, which may mimic infected FOD, presents with diffuse bone destruction and sequestration rather than the well-defined symmetrical masses of FOD. Correct diagnosis is essential to avoid unnecessary and potentially dangerous interventions [3,8].

Treatment for FOD depends on the symptoms. Asymptomatic patients do not require surgery but benefit from regular radiographic surveillance, which can last up to three years, and excellent oral hygiene to prevent secondary infection. In symptomatic cases, such as this patient's, in the absence of consensus on the most effective treatment, a conservative management plan is preferred, including antibiotics and limited excision if necessary. Given the high risk of complications associated with invasive procedures, aggressive surgical approaches are avoided [9]. With regard to the antibiotics to be prescribed, reviews of the literature have suggested that an infected FOD should be treated as osteomyelitis.

## Conclusions

The diagnosis of florid cemento-osseous dysplasia of the jaw is usually made on the basis of clinical findings and radiographic features. However reassuring a biopsy may be, in this situation it may be the trigger for an infection that is difficult to control without major surgery. A conservative approach is always preferable, although therapeutic management will depend on the patient's symptoms.
